# The EntOptLayout Cytoscape plug-in for the efficient visualization of major protein complexes in protein–protein interaction and signalling networks

**DOI:** 10.1093/bioinformatics/btz257

**Published:** 2019-04-20

**Authors:** Bence Ágg, Andrea Császár, Máté Szalay-Bekő, Dániel V Veres, Réka Mizsei, Péter Ferdinandy, Péter Csermely, István A Kovács

**Affiliations:** 1 Department of Pharmacology and Pharmacotherapy, Semmelweis University, 1428 Budapest, Hungary; 2 Heart and Vascular Center, Semmelweis University, 1122 Budapest, Hungary; 3 Pharmahungary Group, 6722 Szeged, Hungary; 4 Department of Medical Chemistry, Semmelweis University, 1428 Budapest, Hungary; 5 Earlham Institute Norwich Research Park, Norwich NR4 7UZ, UK; 6 Turbine Ltd, 1136 Budapest, Hungary; 7 Laboratory of Immunobiology, Department of Medical Oncology, Dana Farber Cancer Institute, Boston, MA 02215, USA; 8 Network Science Institute, Northeastern University, Boston, MA 02115, USA; 9 Center for Cancer Systems Biology (CCSB) and Department of Cancer Biology, Dana-Farber Cancer Institute, Boston, MA 02215, USA; 10 Wigner Research Centre for Physics, Institute for Solid State Physics and Optics, 1525 Budapest, Hungary

## Abstract

**Motivation:**

Network visualizations of complex biological datasets usually result in ‘hairball’ images, which do not discriminate network modules.

**Results:**

We present the EntOptLayout Cytoscape plug-in based on a recently developed network representation theory. The plug-in provides an efficient visualization of network modules, which represent major protein complexes in protein–protein interaction and signalling networks. Importantly, the tool gives a quality score of the network visualization by calculating the information loss between the input data and the visual representation showing a 3- to 25-fold improvement over conventional methods.

**Availability and implementation:**

The plug-in (running on Windows, Linux, or Mac OS) and its tutorial (both in written and video forms) can be downloaded freely under the terms of the MIT license from: http://apps.cytoscape.org/apps/entoptlayout.

**Supplementary information:**

Supplementary data are available at Bioinformatics online.

## 1 Introduction

Informative network layouts enable us an intuitive, direct, qualitative understanding of complex systems preceding more elaborate quantitative studies (Hu and Nöllenburg, 2016; Miryala *et al.*, 2017). Recent contributions to network representation (like Dehmamy *et al.*, 2018; McInnes *et al.*, 2018; Muscoloni *et al.*, 2017) may provide additional approaches in the future. The widely used Cytoscape program has several useful network visualization tools (Shannon *et al.*, 2003). Modular organization is especially informative in interactomes and signalling networks, where network modules represent major protein complexes offering an intuitive insight of their functions (Fessenden, 2017; King *et al.*, 2004; Szalay-Bekő *et al.*, 2012). However, existing network visualization methods lack an information theoretic foundation, and often result in ‘hairball’ images, which are unable to discriminate network modules. Here, we introduce the EntOptLayout Cytoscape plug-in, which uses the novel, relative entropy minimization-based network representation method we developed earlier (Kovács *et al.*, 2015). This method introduces network nodes as probability distributions and selects their best spatial representation, which is the hardest to distinguish from the input data. This is achieved by minimizing the relative entropy (also known as the Kullback-Leibler divergence) between the input data and their representation (Kovács *et al.*, 2015). The EntOptLayout plug-in is able to visualize network modules, highlighting major protein and signalling complexes.

## 2 Materials and methods

The EntOptLayout Cytoscape plug-in initializes the layout using user provided or random coordinates by assigning a Gaussian probability distribution to each node. The relationships between the nodes are then captured by pairwise overlaps of the node distributions. For a network of *n* nodes and *e* edges, the runtime complexity of the plug-in is *∼O(n^2^)* (Kovács *et al.*, 2015). The layout is updated in a user-selected frequency to see partial results, while an adjustable time limit is also available. EntOptLayout has several optimization features and user-friendly options as detailed in the Supplementary Data and its Tutorial. As an important option, EntOptLayout may raise the adjacency matrix on the square, which captures the interaction profile similarity of the nodes, and improves the detection of functional network modules even further. EntOptLayout is compatible with Cytoscape 3.7.1 and will be upgraded to its later versions. The source code of the plug-in can be accessed and support tickets can be issued here: https://sourceforge.net/projects/entopt/. Between January 2017 and February 2019 the plug-in was downloaded more than 4700 times and received only maximal, 5-star evaluations.

## 3 Results and conclusion

**Fig. 1.**
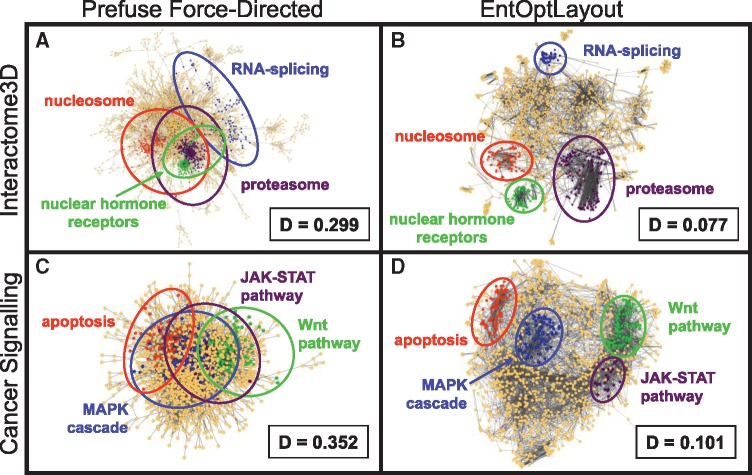
Visualization of major protein complexes by the EntOptLayout Cytoscape plug-in. Coloured segments of the image represent various major protein complexes, showing the same, maximum 200 core nodes of the respective network module/community identified by the ModuLand Cytoscape plug-in (Szalay-Bekő *et al.*, 2012) as detailed in the legend of Supplementary Figure S1. Panels **A** and **B** show the Interactome3D human protein–protein interaction network (Mosca *et al.*, 2013) visualized by the Cytoscape prefuse force-directed layout option alone or by the subsequent use of the EntOptLayout plug-in (switching on the square of the adjacency matrix, ignoring the square of the diagonal and performing consecutive optimizations for 10 000 s each for node position, node width, node position, node width and node position after a pre-ordering made by the prefuse force directed layout), respectively. ‘D’ denotes the normalized information loss (relative entropy) of the layouts stored in the Network Table of the plug-in (in case of the Cytoscape layout its node positions were imported to the EntOptLayout plug-in, and only the node probability distributions were optimized keeping the node positions intact as described in Chapter 5 of the plug-in Tutorial). We note that the 10 000 s alternating position and node width optimization steps should be continued until the ‘D’ value (the normalized information loss) of the layout is decreasing. ‘D’ values are usually becoming minimal after 4–5 subsequent optimization steps. We also note that the use of the prefuse force-directed layout as a pre-ordering layout option before the use of the EntOptLayout shortens the required optimization time and allows the correct positioning of a few (usually 1–4) nodes, which became mis-positioned if this pre-ordering is not used. We recommend the use of the prefuse force-directed algorithm as pre-ordering, since the combination of only this algorithm with the EntOptLayout (but not 3 other Cytoscape layout options) resulted the correct positioning of all nodes (see Supplementary Fig. S2). Panels **C** and **D** show the map of human cancer signalling (Cui *et al.*, 2007) visualized in the same way as shown in Panels A and B

Figure 1A and B show the Interactome3D human protein–protein interaction network (Mosca *et al.*, 2013), visualized by the prefuse force-directed layout option of Cytoscape (Fig. 1A) or by the subsequent use of the EntOptLayout plug-in (Fig. 1B). While the core of the standard Cytoscape visualization was a typical ‘hairball’ image, where protein complexes had a large overlap, EntOptLayout using the ‘square of adjacency matrix’ option displayed the major protein complexes as distinct visual subgroups of the interactome. The same, or even larger differences were observed comparing various other standard Cytoscape and EntOptLayout images, and examining the Cytoscape example genetic interaction, human disease and 75 top node STRING Alzheimer's disease-related interactome network, as well as weighted normal or heat shocked yeast BioGrid interactomes, the map of human cancer signalling, the Reactome human pathway network or network modules of benchmark graphs (see Fig. 1C and D; Supplementary Data; Supplementary Figs S1–S9 and S12–S14). In case of the affinity purification and 500 top node STRING network (Supplementary Figs S10 and S11) all the four visualizations (original, prefuse force-directed, spring embedded and EntOptLayout) showed significant overlaps of the modules. On the contrary, modules were clearly distinct and well separated on the EntOptLayout image in case of all the nine other networks listed above, while they showed significant overlaps when original layouts, spring embedded layouts, prefuse force-directed layouts or other layout options were examined (Fig. 1; Supplementary Figs S1–S9 and S12–S14). Importantly, the normalized information loss (relative entropy, Kullback-Leibler divergence) between the input data and their layout representation showed a 3- to 25-fold improvement when the EntOptLayout method was compared to conventional methods in all cases examined (Fig. 1 and Supplementary Figs S1–S14).

Interestingly, the edge structure of the spring embedded layout also showed dense clusters, which may imply a modular structure. However, this modular structure became covered if the diameter of the nodes was increased to a usual size. On the contrary, the same modular structure remained clearly identifiable when we used the EntOptLayout, since this latter algorithm gave a distinct localization of the modules. Such distinct localization could not be observed when using the spring embedded layout (Supplementary Fig. S3).

In summary, we highlight the ‘pros’ and ‘cons’ of using the EntOptLayout network visualization Cytoscape plugin. The major advantage of using EntOptLayout is that it is the only algorithm, which gives a clear visual discrimination of functional protein complexes in most networks. Better optical discrimination of protein complexes may help to discover the emergence of novel functions in changing interactomes or signalling networks during the propagation of a disease, cellular differentiation, wound healing, embryogenesis, etc. Importantly, the algorithm also minimizes the information loss during the visualization process, thus its image is not only functionally better but is also theoretically closer to an ‘optimal’ image. It is a disadvantage of the EntOptLayout algorithm that it sometimes—mostly when using the ‘square of adjacency matrix option’—gives aesthetically less pleasing images than other visualization algorithms, such as the widely used force-directed algorithm. This is due to the fact that the EntOptLayout does not optimize the image for the shortest length of edges or for crossing edges. An additional disadvantage of the EntOptLayout in case of large networks is the 10 000 s suggested running time of each optimization cycle as described in the legend of Figure 1. We are currently developing an upgrade of the algorithm which will allow shorter running times.

In conclusion, the use of the EntOptLayout plug-in in 9 out of 11 cases outperformed alternative Cytoscape layout options in the visual discrimination of network modules. This is especially important in human interactomes and signalling networks, providing an intuitive insight into the functional organization under healthy and pathological conditions.

## Supplementary Material

btz257_Supplementary_Data
