# Supplementary material for: The EntOptLayout Cytoscape plug-in for the efficient visualization of major protein complexes in protein–protein interaction and signalling networks
Source: Bioinformatics. 2019 Apr 20;35(21):4490–2. doi: 10.1093/bioinformatics/btz257 (PMC6821346; doi:10.1093/bioinformatics/btz257)
Supplement: btz257_Supplementary_Data [file btz257_supplementary_data.pdf]

Supplementary Data

The EntOptLayout Cytoscape plug-in for the efficient visualization of major protein complexes in protein-protein interaction and signalling networks

Bence Ágg[1-3], Andrea Császár[4], Máté Szalay-Bekő[4,5], Dániel V. Veres[4,6], Réka Mizsei[7], Péter Ferdinandy[1,3], Péter Csermely[4,*] and István A. Kovács[8-10]

[1]Department of Pharmacology and Pharmacotherapy, Semmelweis University, Budapest, 1428 Hungary, [2]Heart and Vascular Center, Semmelweis University, 1122 Budapest, Hungary, [3]Pharmahungary Group, 6722 Szeged, Hungary, [4]Department of Medical Chemistry, Semmelweis University, Budapest, 1428 Hungary, [5]Earlham Institute, Norwich Research Park, Norwich, NR4 7UZ, UK, [6]Turbine Ltd, Budapest, 1136 Hungary, [7]Laboratory of Immunobiology, Department of Medical Oncology, Dana Farber Cancer Institute Boston, 02215 MA, USA, [8]Network Science Institute, Northeastern University, 02115 Boston MA, USA, [9]Center for Cancer Systems Biology (CCSB) and Department of Cancer Biology, Dana-Farber Cancer Institute, Boston, MA 02215, USA, [10]Wigner Research Centre for Physics, Institute for Solid State Physics and Optics, Budapest, 1525 Hungary

*To whom correspondence should be addressed. E-mail: csermely.peter@med.semmelweis-univ.hu

Contents

Supplementary Methods 3
Supplementary Results 4
Supplementary Figures
Supplementary Figure S1. Comparison of the Interactome3D human protein-protein interaction network layout (A,B) and the map of human cancer signalling (C,D) made by the EntOptLayout plug-in with (A,C) or without (B,D) Cytoscape prefuse force-directed pre-ordering........ 5
Supplementary Figure S2. Comparison of the Interactome3D human protein-protein interaction network layout made by the EntOptLayout plug-in using Cytoscape force directed (A), spring-embedded (B), circular layout (C) and prefuse force-directed pre-ordering (D). 6
Supplementary Figure S3. Comparison of the visually detectable modular densities of the Interactome3D human protein-protein interaction network layout made by the Cytoscape spring embedded layout (A and B) and the EntOptLayout plug-in using Cytoscape force directed pre-ordering (C and D). 7
Supplementary Figure S4. Comparison of the Interactome3D human protein-protein interaction network layout made by the prefuse force-directed option of Cytoscape without (A) or with the square of the adjacency matrix (B) and the EntOptLayout plug-in without (C) or with (D) the square of the adjacency matrix........ 8
Supplementary Figure S5. Comparison of the Interactome3D human protein-protein interaction network layout made by the prefuse force-directed option of Cytoscape (A), the EntOptLayout (B), the spring embedded layout of Cytoscape (C) and the EClerize plug-in (D). 9
Supplementary Figure S6. Comparison of the normal and heat shocked yeast interactome layouts made by the prefuse force-directed option of Cytoscape and the EntOptLayout plug-in. 10
Supplementary Figure S7. Comparison of benchmark graph layouts made by the prefuse force-directed option of Cytoscape (panels A and C) and the EntOptLayout plug-in (Panels B and D). 11

Contents is continued on the next page

Contents (continued from the previous page)

Supplementary Figures (continued from the previous page)

Supplementary Figure S8. Comparison of the Reactome human pathway network layout made by the prefuse force-directed option of Cytoscape (A) and the EntOptLayout plug-in (B). 12

Supplementary Figure S9. Comparison of the map of human cancer signalling (A,B) and the Reactome human pathway network (C,D) EntOptLayout made with (A,C) or without the adaptor/scaffold protein interactions (B,D). 13

Supplementary Figure S10. Comparison of the Cytoscape welcome screen affinity purification example network original layout (A), prefuse force directed layout (B), spring-embedded layout (C) and EntOptLayout (D). 14

Supplementary Figure S11. Comparison of the Cytoscape welcome screen genetic interaction example network original layout (A), prefuse force directed layout (B), spring-embedded layout (C) and EntOptLayout (D). 15

Supplementary Figure S12. Comparison of the Cytoscape welcome screen diseases example network original layout (A), prefuse force directed layout (B), spring-embedded layout (C) and EntOptLayout (D). 16

Supplementary Figure S13. Comparison of the 75 node STRING Alzheimer's disease-related protein-protein interaction network original layout (A), prefuse force directed layout (B), spring-embedded layout (C) and EntOptLayout (D). 17

Supplementary Figure S14. Comparison of the 500 node STRING Alzheimer's disease-related protein-protein interaction network original layout (A), prefuse force directed layout (B), spring-embedded layout (C) and EntOptLayout (D). 18

Supplementary References 19

Supplementary Methods

The EntOptLayout Cytoscape plug-in (downloadable freely under the terms of the MIT license from: http://apps.cytoscape.org/apps/entoptlayout; its source code available and support tickets can be issued here: https://sourceforge.net/projects/entopt/) starts the layout from either user provided or random coordinates by assigning a Gaussian probability distribution to each node. The relationships between the nodes are then captured by pairwise overlaps of the node distributions (Kovács *et al.*, 2015). To minimize the memory requirements, computation of the overlaps are performed 'on-the-fly', when they are needed, without a significant performance loss. For the same reason, adjacency matrices are stored using the Compressed Row Storage sparse matrix representation (Saad, 2003). Besides node positions, the height and width of node probability distributions can also be optimized in separate Newton-Raphson iteration steps to minimize the relative entropy. If applied in an alternating fashion these iteration steps improve the quality of the achieved network layout substantially.

EntOptLayout has several additional user-friendly options. Diagonal elements of the adjacency and overlap matrices can be ignored or included once or twice (for both directions) depending on the ignorance or emphasis on self-loops. Consideration of edge weights is also optional.

The installation of the plug-in follows Cytoscape procedures. EntOptLayout can be distributed as a single .jar file working on Linux, Windows and Mac OS with the respective Cytoscape versions. The plug-in has a detailed step-by-step tutorial, whose written and video forms can be downloaded from the plug-in's webpage: http://apps.cytoscape.org/apps/entoptlayout.

Besides the layout image EntOptLayout exports the optimized 2D positions, widths and heights of node probability distributions and provides both the summarized information loss and its normalized version (the relative entropy, D) of the network. The plug-in is also able to order the input network data by optimizing node positions in one dimension, exporting the ordered dataset as a conventional spreadsheet.

Supplementary Results

The major advance of the EntOptLayout plugin is, that on the contrary to standard Cytoscape visualizations, which resulted in a typical 'hairball' image (where protein complexes had a large overlap), the EntOptLayout using the 'square of adjacency matrix' option displayed the major protein complexes as distinct visual subgroups. This was first demonstrated by visualizing the Interactome3D human protein-protein interaction network (*n*=3031, *e*=5772, Mosca *et al*., 2013; see Fig. 1. of the main text).

The same marked differences persisted, when we compared to other standard tools, such as to spring-embedded (D=0.294), yFiles organic (D=0.369) or self-organizing-map (D=0.572) Cytoscape layout. Similarly large, albeit smaller differences were observed, when we used the EntOptLayout plug-in without the 'square of adjacency matrix' option compared with standard Cytoscape or EClerize plug-in (Danaci, 2015) layouts, as well as when we gave the square of adjacency matrix as an input to standard Cytoscape layouts. Importantly, when using random-seeds instead of prefuse force-directed pre-ordering, 3 nodes of the marked approx. 1000 nodes of the Interactome3D became overlapping with other modules (see asterisks on Supplementary Fig. 1B) and a variable overlap occurred in case of other datasets (having zero to 20 overlapping nodes from the marked approx. thousand total; Supplementary Fig. 1D and data not shown). This is due to the unfavourable random starting localization of a few nodes as we discussed in our original paper (Kovács et al, 2015; page 4). This is why we regularly used the Cytoscape prefuse force-directed pre-ordering in our visualizations and suggest to use it to the users of the EntOptLayout plug-in as an input (Supplementary Figs. S1, S2 and S3 and data not shown).

The same, or even larger differences were observed, when comparing standard Cytoscape and EntOptLayout images of the normal or heat shocked yeast BioGrid interactomes (*n*=5,223, *e*=44,314 weighted edges, Supplementary Fig. S4). Here, the marked increase in the yeast interactome modularity after heat shock (Mihalik and Csermely, 2011) was seen only on the EntOptLayout image (Supplementary Fig. S4). Here again, the marked differences persisted upon comparing the EntOptLayout 'square of adjacency matrix' option with standard Cytoscape layouts using the square of the adjacency matrix as an input (data not shown).

These observations were supported further by the clear discrimination of network modules of the benchmark graphs of Lancichinetti *et al.* (2008) using the EntOptLayout plug-in, in contrast to standard Cytoscape layout options (Supplementary Fig. S5).

Visualization of the map of human cancer signalling (*n*=1,609, *e*=5,049, Cui *et al*., 2007) gave similar differences between standard Cytoscape layout options (Fig. 1C of the main text, Supplementary Fig. S2 and data not shown) and the EntOptLayout plug-in (Fig. 1D of the main text, Supplementary Fig S1). We found a clear visual discrimination of major cancer related signalling pathways only in the latter case. Similar, marked differences were observed, when the Reactome human pathway network (*n*=6825, *e*=17,529, Croft *et al*., 2011) was visualized (Supplementary Fig. S6). Of note, the modular organization was more pronounced when protein-protein interactions were included to these two datasets (Supplementary Fig. S7). The weaker modular organization of kinases and their substrate proteins alone was confirmed by the EntOptLayout visualization of the human PhosphositePlus network (*n*=2558, *e*=10,145, Hornbeck *et al*., 2015), a dataset lacking protein-protein interactions (data not shown). Importantly, the normalized information loss (relative entropy, Kullback-Leibler divergence) between the input data and their layout representation showed a 3- to 13-fold improvement when the EntOptLayout method was compared to conventional methods (Fig. 1 and Supplementary Figs. S1 to S7).

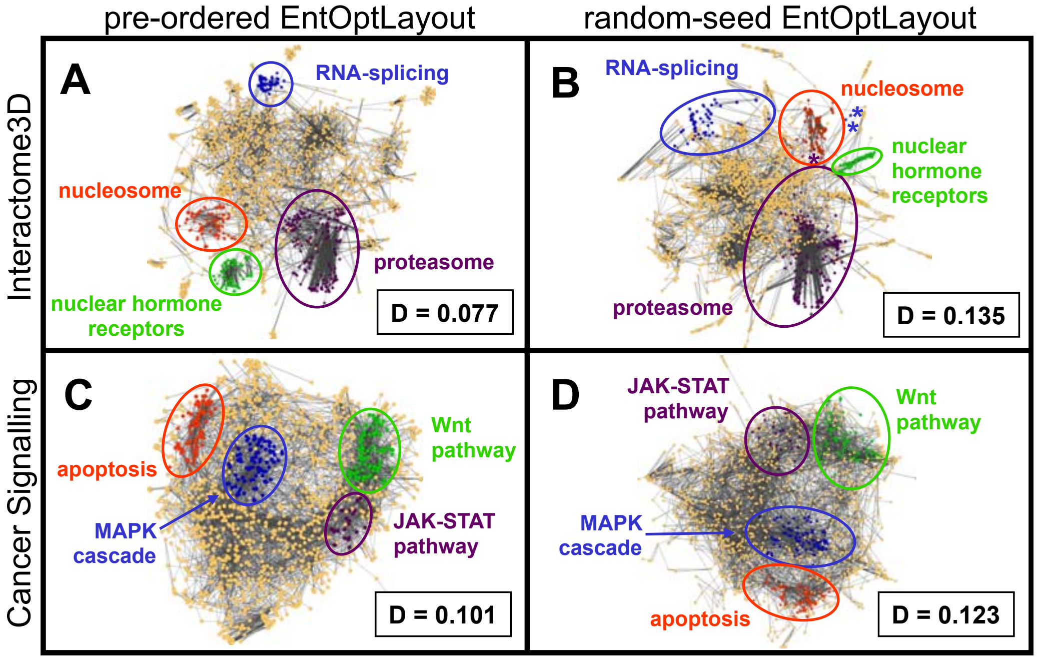

Supplementary Figure S1. Comparison of the Interactome3D human protein-protein interaction network layout (A,B) and the map of human cancer signalling (C,D) made by the EntOptLayout plug-in with (A,C) or without (B,D) Cytoscape prefuse force-directed pre-ordering. Panels A and B show the Interactome3D human protein-protein interaction network (Mosca *et al.*, 2013). Panels C and D show the map of human cancer signalling (Cui et al., 2007). Networks were visualized by the EntOptLayout Cytoscape plug-in either after a pre-ordering of the nodes using the Cytoscape (Shannon *et al.*, 2003) prefuse force-directed layout option (Panels A and C) or starting from random seeds (Panels B and D) using default settings switching on 'the second power of the adjacency matrix' option of the EntOptLayout plug-in and the same settings as detailed in the legend of Fig 1. of the main text. “D” denotes the normalized information loss (relative entropy) of the layouts. Major protein complexes were identified by the consensus function of their nodes having the largest community centrality (showing the same, maximum 200 core nodes of the respective network community as calculated by the ModuLand plug-in; Szalay-Bekő *et al.,* 2012), as well as by identifying the consensus functions of the majority of marked nodes in Uniprot (The UniProt Consortium, 2017). The size of each group was determined until a minimum of the community centrality values was found marking the border between two adjacent network modules (Kovács *et al.*, 2010; Szalay-Bekő *et al.,* 2012). Circled segments of the image highlight various major protein complexes (Panels A and B: blue: RNA-splicing and maturation; purple: proteasome; green: nuclear hormone receptors and red: nucleosome + related proteins; Panels C and D: blue: MAPK cascade; purple: JAK-STAT pathway; green: Wnt pathway and red: apoptosis). As it is shown in Panels B and D starting from random seeds resulted in higher D values, which were still (much) less than half of the D-values of the Cytoscape force-directed layout alone (0.135 instead 0.299 and 0.123 instead of 0.352, in case of Interactome3D and map of human cancer signalling, respectively). Importantly, at the Interactome3D layout one node of the proteasome-complex and two nodes of the RNA-splicing complex were overlapping with other modules (see purple and blue asterisks of Panel B, respectively). This is due to the unfavourable starting localization of a few nodes, as we discussed in our previous paper (Kovács et al, 2015, page 4). Optimizing to 6 to 9 times more of the original 10,000 sec run reached similar D values than those obtained at the pre-ordered case (Panels A and C). However, the layout still had a similarly low number (zero to 4) outlier nodes, which were differing if the random seed was changed (data not shown).

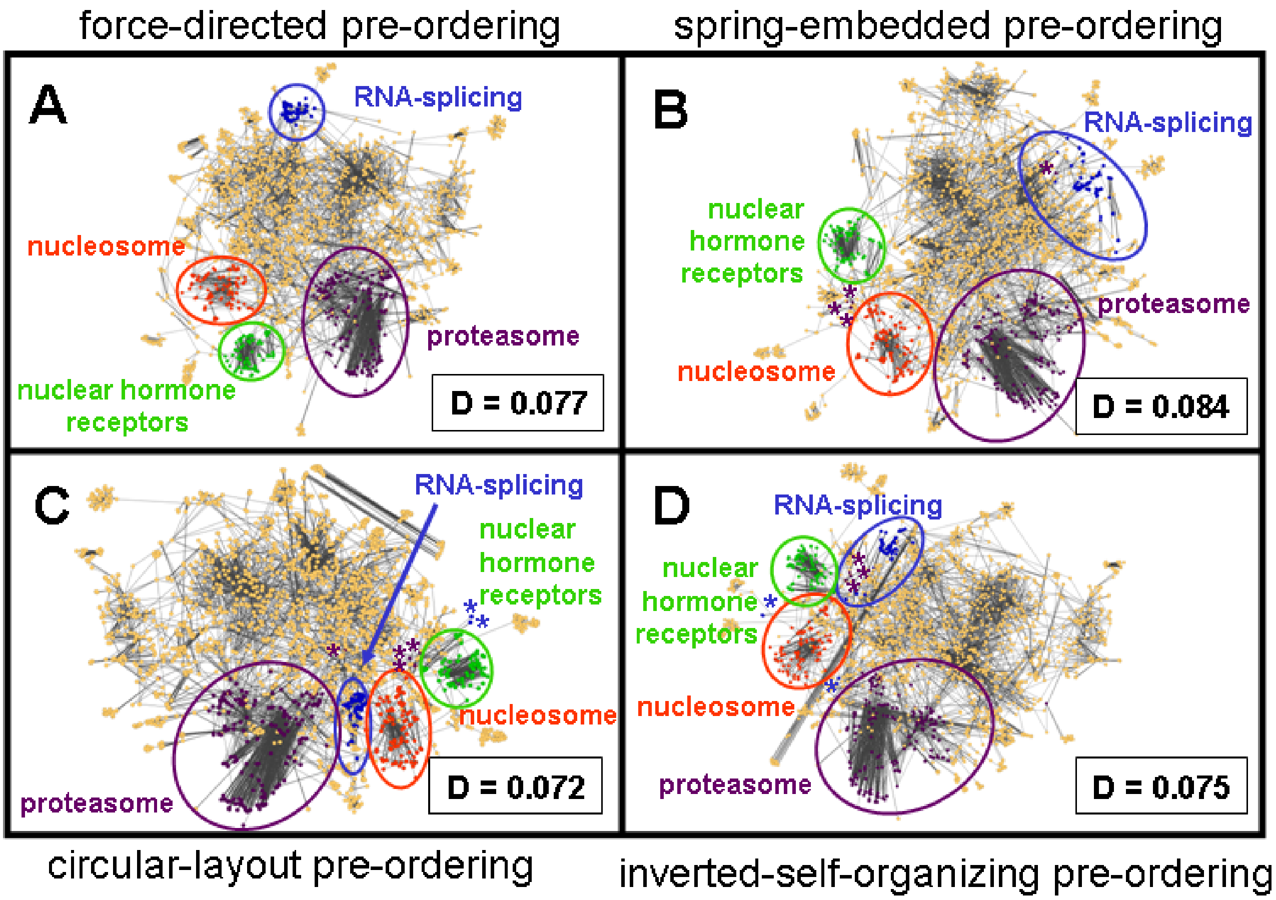

Supplementary Figure S2. Comparison of the Interactome3D human protein-protein interaction network layout made by the EntOptLayout plug-in using Cytoscape force directed (A), spring-embedded (B), circular layout (C) and prefuse force-directed pre-ordering (D). The figure shows the Interactome3D human protein-protein interaction network (Mosca *et al.*, 2013) visualized by the EntOptLayout Cytoscape plug-in either after a pre-ordering of the nodes using the Cytoscape (Shannon *et al.*, 2003) force directed (Panel A), spring-embedded (Panel B), circular layout (Panel C) and prefuse force-directed pre-ordering options (Panel D). In the visualization process we used default settings switching on 'the second power of the adjacency matrix' option of the EntOptLayout plug-in and the same settings as detailed in the legend of Fig 1. of the main text. "D" denotes the normalized information loss (relative entropy) of the layouts. Major protein complexes were identified by the consensus function of their nodes having the largest community centrality (showing the same, maximum 200 core nodes of the respective network community as calculated by the ModuLand plug-in; Szalay-Bekő *et al.*, 2012), as well as by identifying the consensus functions of the majority of marked nodes in Uniprot (The UniProt Consortium, 2017). The size of each group was determined until a minimum of the community centrality values was found marking the border between two adjacent network modules (Kovács *et al.*, 2010; Szalay-Bekő *et al.*, 2012). Circled segments of the image highlight various major protein complexes (blue: RNA-splicing and maturation; purple: proteasome; green: nuclear hormone receptors and red: nucleosome + related proteins). Though the D values did not differ much using various pre-ordering functions (however, all were significantly smaller than that of the random-seed layout; D=0.135, see panel B of Supplementary Figure S1), only force directed pre-ordering resulted in zero outlier nodes (see Panel A), while with each of the other three pre-ordering there were 4, 5 and 5 outlier nodes on Panels B, C and D, respectively.

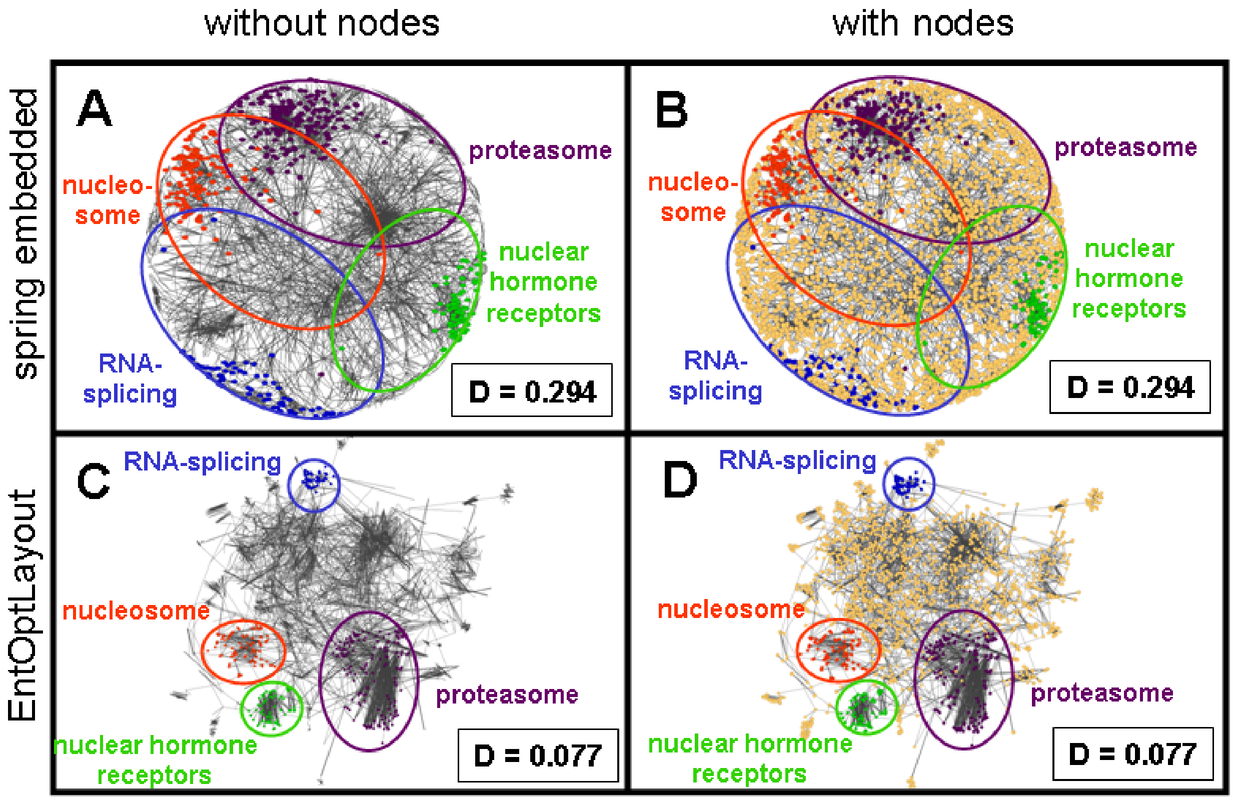

Supplementary Figure S3. Comparison of the visually detectable modular densities of the Interactome3D human protein-protein interaction network layout made by the Cytoscape spring embedded layout (A and B) and the EntOptLayout plug-in using Cytoscape force directed pre-ordering (C and D). The figure shows the Interactome3D human protein-protein interaction network (Mosca *et al*., 2013) visualized by the Cytoscape (Shannon *et al*., 2003) spring embedded layout (Panels A and B) or the EntOptLayout Cytoscape plug-in after pre-ordering with the Cytoscape (Shannon *et al*., 2003) prefuse force-directed pre-ordering option (Panels C and D). In the visualization process we used default settings switching on 'the second power of the adjacency matrix' option of the EntOptLayout plug-in and the same settings as detailed in the legend of Fig 1. of the main text. “D” denotes the normalized information loss (relative entropy) of the layouts (in case of the spring embedded layout its node positions were imported to the EntOptLayout plug-in, and only the node probability distributions were optimized keeping the node positions intact). Major protein complexes were identified by the consensus function of their nodes having the largest community centrality (showing the same, maximum 200 core nodes of the respective network community as calculated by the ModuLand plug-in; Szalay-Bekő *et al.,* 2012), as well as by identifying the consensus functions of the majority of marked nodes in Uniprot (The UniProt Consortium, 2017). The size of each group was determined until a minimum of the community centrality values was found marking the border between two adjacent network modules (Kovács *et al*., 2010; Szalay-Bekő *et al.,* 2012). Circled segments of the image highlight various major protein complexes (blue: RNA-splicing and maturation; purple: proteasome; green: nuclear hormone receptors and red: nucleosome + related proteins). Though the edge densities of both the spring embedded layout (Panel A) and EntOptLayout options (Panel C) reveal some modules the EntOptLayout modules are visually more distinct, therefore they remain detectable if we increase the node size (Panel D). Note that the visually detectable modular densities of the spring embedded layout shown on Panel A became largely covered if we increase the node size as shown on Panel B.

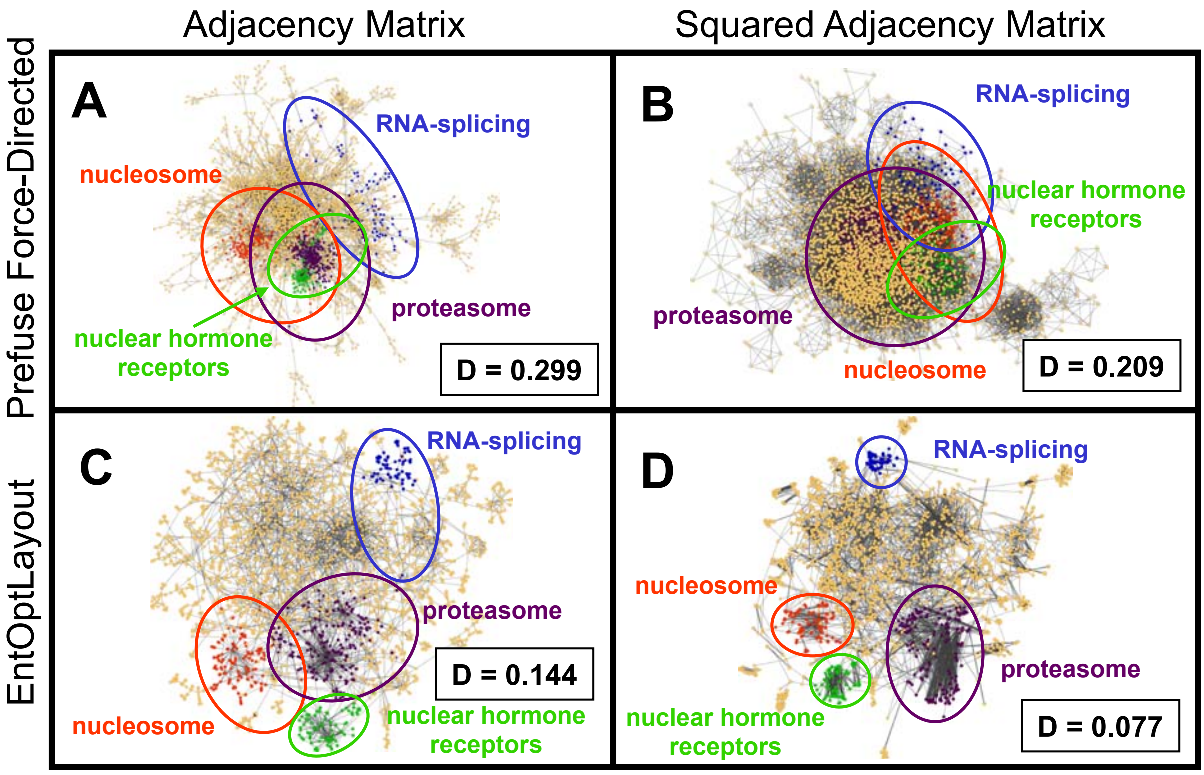

Supplementary Figure S4. Comparison of the Interactome3D human protein-protein interaction network layout made by the prefuse force-directed option of Cytoscape without (A) or with the square of the adjacency matrix (B) and the EntOptLayout plug-in without (C) or with (D) the square of the adjacency matrix. Panels A through D show the Interactome3D human protein-protein interaction network (Mosca *et al*., 2013) visualized by the Cytoscape (Shannon *et al*., 2003) prefuse force-directed layout option alone without (Panel A) or with the square of the adjacency matrix of the network as an input, and by the subsequent use of the EntOptLayout plug-in using the same settings as detailed in the legend of Fig 1. of the main text without (Panel C) and with (Panel D) the 'square of the adjacency matrix' option. "D" denotes the normalized information loss (relative entropy) of the layouts (in case of the Cytoscape layout its node positions were imported to the EntOptLayout plug-in, and only the node probability distributions were optimized keeping the node positions intact). Circled segments of the image highlight various major protein complexes (blue: RNA-splicing and maturation; purple: proteasome; green: nuclear hormone receptors and red: nucleosome + related proteins). Major protein complexes were identified by the consensus function of their nodes having the largest community centrality (showing the same, maximum 200 core nodes of the respective network community as calculated by the ModuLand plug-in; Szalay-Bekő *et al.,* 2012).

\

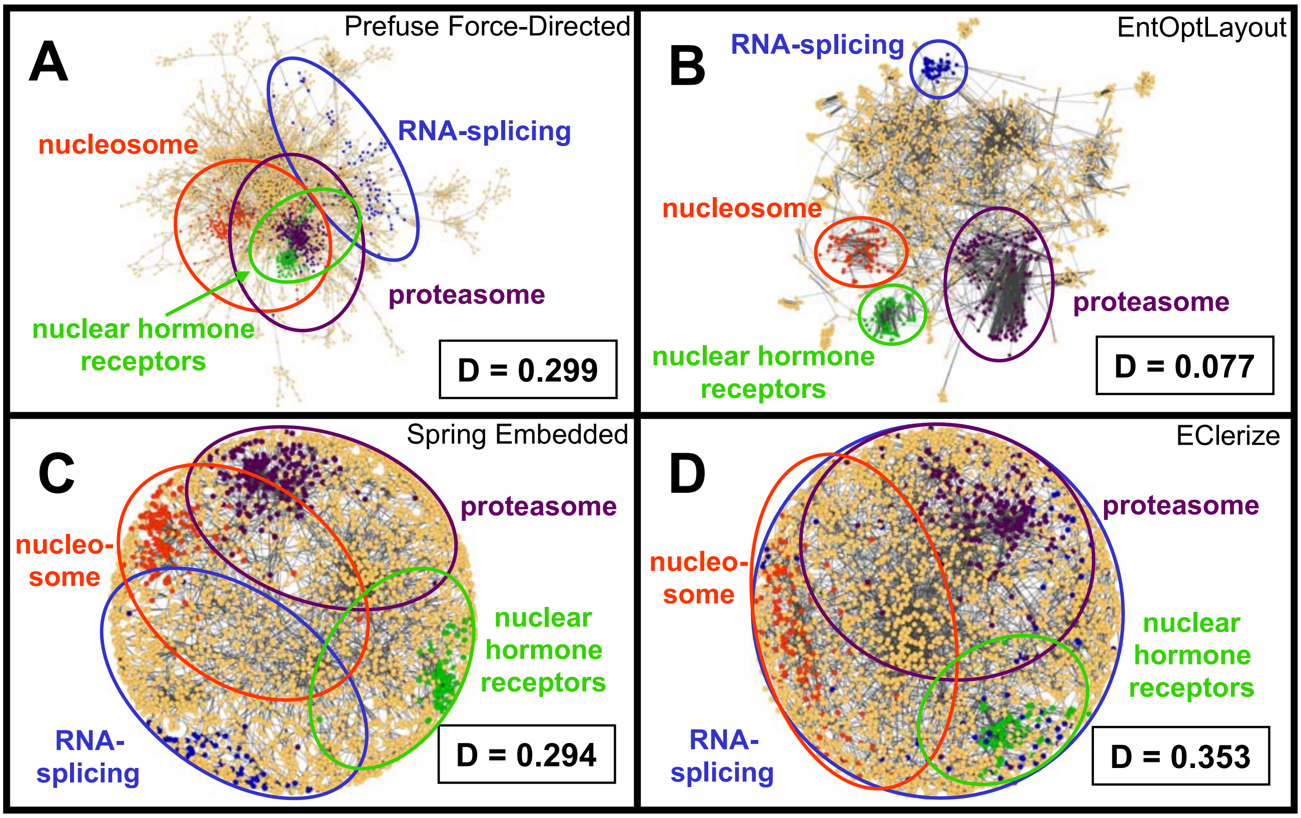

Supplementary Figure S5. Comparison of the Interactome3D human protein-protein interaction network layout made by the prefuse force-directed option of Cytoscape (A), the EntOptLayout (B), the spring embedded layout of Cytoscape (C) and the EClerize plug-in (D). Panels A through D show the Interactome3D human protein-protein interaction network (Mosca *et al*., 2013) visualized by the Cytoscape (Shannon *et al*., 2003) prefuse force-directed layout option alone (Panel A), by the subsequent use of the EntOptLayout plug-in using the same settings as detailed in the legend of Fig 1. of the main text with the 'square of the adjacency matrix' option (Panel B), the spring-embedded Cytoscape layout (Panel C) and the EClerize plug-in (Panel D; Danaci, 2015). When using the EClerize plug-in the 'number of layout passes' was set to 150 instead of the default value of 10 to ensure the best alignment. "D" denotes the normalized information loss (relative entropy) of the layouts (in case of the Cytoscape and EClerize layouts their node positions were imported to the EntOptLayout plug-in, and only the node probability distributions were optimized keeping the node positions intact). Circled segments of the image highlight various major protein complexes (blue: RNA-splicing and maturation; purple: proteasome; green: nuclear hormone receptors and red: nucleosome + related proteins). Major protein complexes were identified by the consensus function of their nodes having the largest community centrality (showing the same, maximum 200 core nodes of the respective network community as calculated by the ModuLand plug-in; Szalay-Bekő *et al.*, 2012).

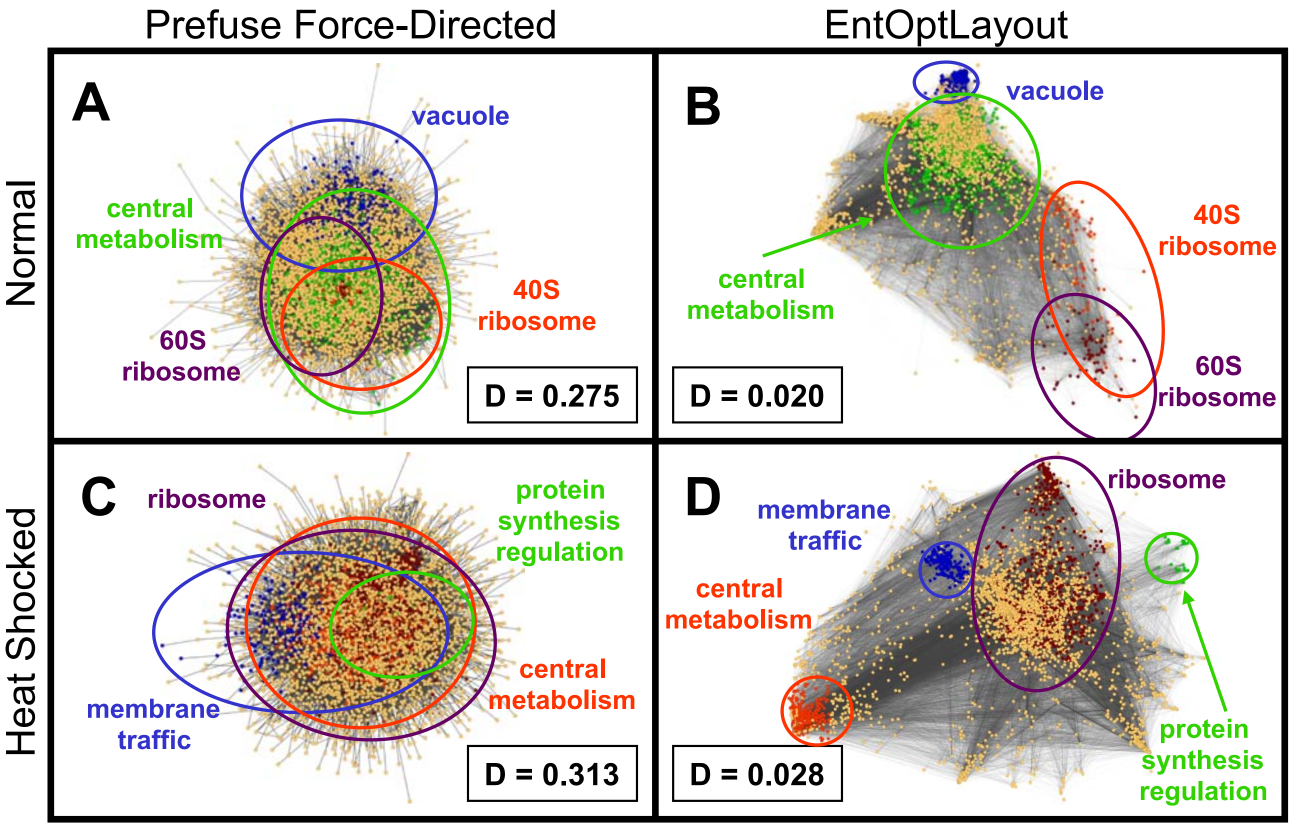

Supplementary Figure S6. Comparison of the normal and heat shocked yeast interactome layouts made by the prefuse force-directed option of Cytoscape and the EntOptLayout plug-in. Panels A and B show the yeast interactome under non-stressed, normal, 30°C conditions (Mihalik and Csermely, 2011) visualized by the Cytoscape (Shannon *et al.*, 2003) prefuse force-directed layout option alone or by the subsequent use of the EntOptLayout plug-in using the same settings as detailed in the legend of Fig 1. of the main text including edge weights with the 'square of the adjacency matrix' option, respectively. Panels C and D show the same two layouts for the yeast interactome exposed to a 15 minutes heat shock on 37°C. The sets of the BioGrid data of 5,223 nodes and 44,314 edges are the same in the two experimental conditions. Edge weights were calculated as interaction probabilities using the product of the node abundances. Node abundances were calculated from the mRNA expression patterns using mRNA abundances as proxies for protein abundance as described earlier (Mihalik and Csermely, 2011). "D" denotes the normalized information loss (relative entropy) of the layouts (in case of the Cytoscape layout its node positions were imported to the EntOptLayout plug-in, and only the node probability distributions were optimized keeping the node positions intact). Circled segments of the image highlight various major protein complexes (Panels A and B: blue, vacuole, 200 nodes; green, central metabolism, 800 nodes; red, 40S ribosome, 81 nodes; purple, 60S ribosome, 63 nodes. Panels C and D: blue membrane traffic, 200 nodes; purple, ribosome, 800 nodes; green, protein synthesis regulation, 25 nodes; red, central metabolism, 400 nodes). Major protein complexes were identified by the consensus function of their nodes having the largest community centrality (showing the same number of core nodes of the respective network community as calculated by the ModuLand plug-in; Szalay-Bekő *et al.*, 2012) identifying the consensus functions of the majority of marked nodes in the Saccharomyces Genome Database (http://yeastgenome.org).

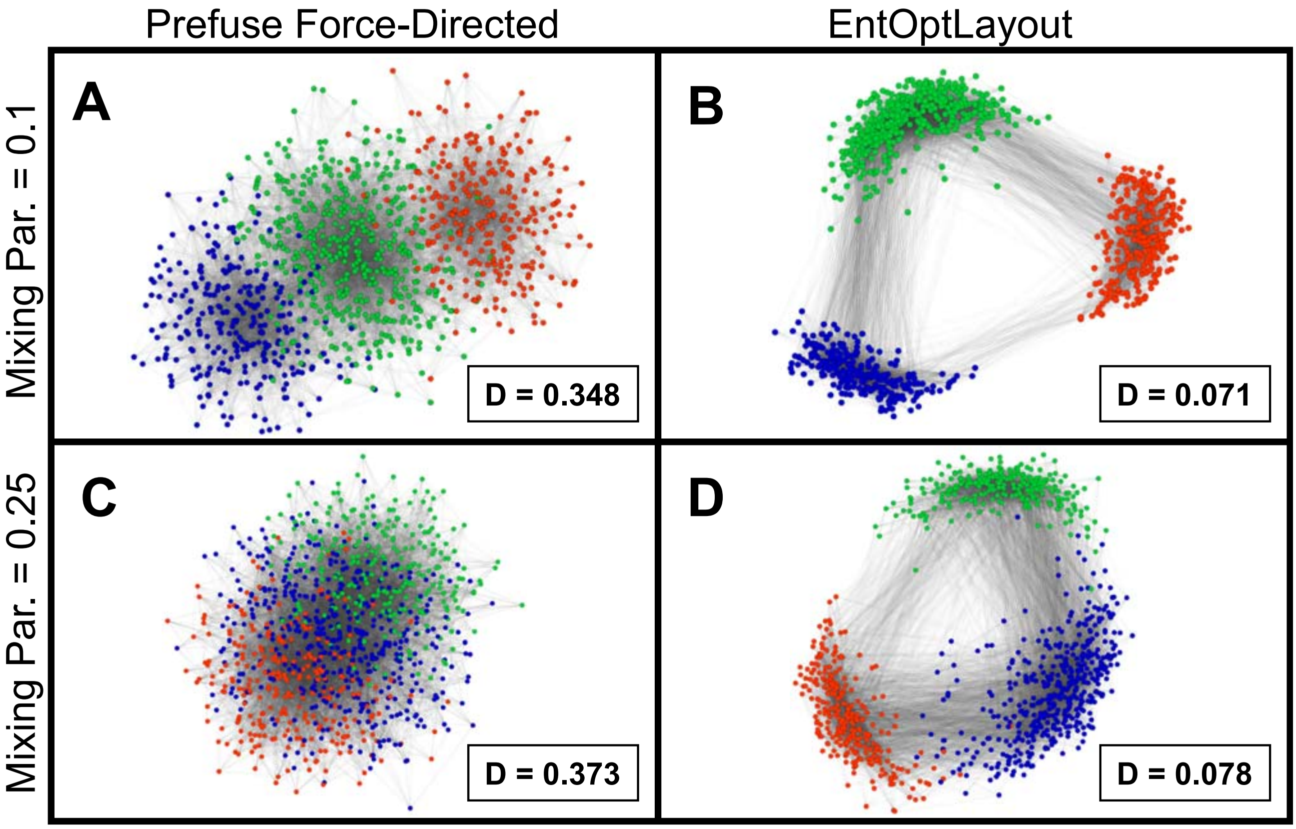

Supplementary Figure S7. Comparison of benchmark graph layouts made by the prefuse force-directed option of Cytoscape (panels A and C) and the EntOptLayout plug-in (Panels B and D). Panels A through D show the scale-free benchmark graphs of Lancichinetti *et al.* (2008) having 3 non-overlapping modules visualized by the Cytoscape (Shannon *et al.*, 2003) prefuse force-directed layout option alone (Panels A and C) or by the subsequent use of the EntOptLayout plug-in using the same settings as detailed in the legend of Fig 1. of the main text with the 'square of the adjacency matrix' option (Panels B and D), respectively. The number of nodes, the average degree, the maximal degree were 1000, 15 and 50, respectively. The mixing parameter (i.e. the parameter whose increase merges the 3 modules of the benchmark graph) was set to 0.1 on Panels A and B, while to 0.25 on Panels C and D. "D" denotes the normalized information loss (relative entropy) of the layouts (in case of the Cytoscape layout its node positions were imported to the EntOptLayout plug-in, and only the node probability distributions were optimized keeping the node positions intact). The 3 modules are barely visible at the mixing parameter of 0.1 using the Cytoscape layout (Panel A), while they are clearly discriminated using the EntOptLayout plug-in (Panel B). None of the 3 modules are visible at the mixing parameter of 0.25 using the Cytoscape layout (Panel C), while they can be still discriminated using the EntOptLayout plug-in (Panel D).

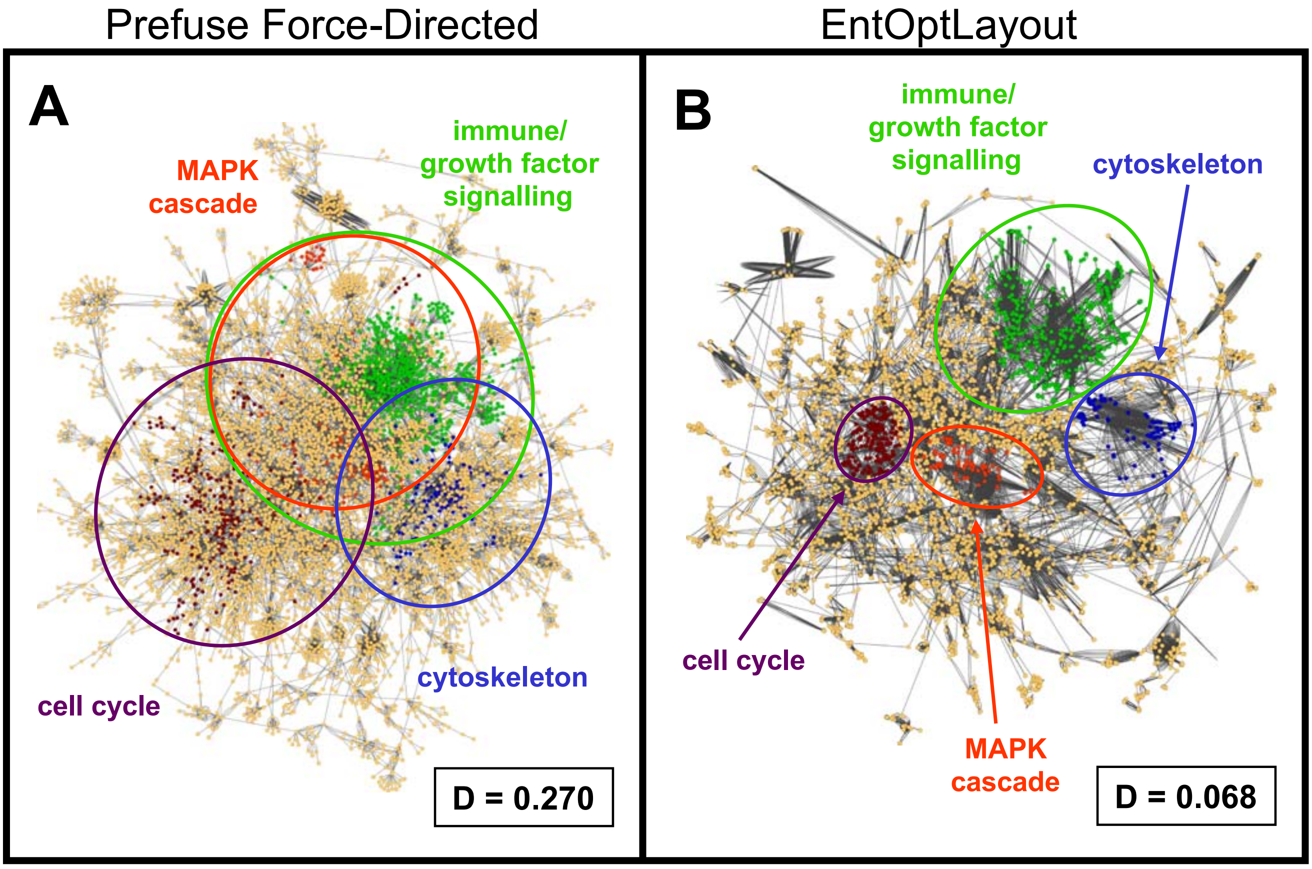

Supplementary Figure S8. Comparison of the Reactome human pathway network layout made by the prefuse force-directed option of Cytoscape (A) and the EntOptLayout plug-in (B). Panels A and B show the Reactome human pathway network (Croft *et al.*, 2011; without self-loops 6825 nodes, 17,529 edges) visualized by the Cytoscape (Shannon *et al.*, 2003) prefuse force-directed layout option alone, or by the subsequent use of the EntOptLayout plug-in using the same settings as detailed in the legend of Fig 1. of the main text with the 'square of the adjacency matrix' option and ignoring self-loops, respectively. “D” denotes the normalized information loss (relative entropy) of the layouts (in case of the Cytoscape layout its node positions were imported to the EntOptLayout plug-in, and only the node probability distributions were optimized keeping the node positions intact). Circled segments of the image highlight various major protein complexes (blue, cytoskeleton, 104 nodes; green, immune/growth factor signalling, 682 nodes; red, MAP kinase pathway, 112 nodes; purple, cell cycle, 249 nodes). Major protein complexes were identified by the consensus function of their nodes having the largest community centrality (showing the same core nodes of the respective network community as calculated by the ModuLand plug-in; Szalay-Bekő *et al.*, 2012) identifying the consensus functions of the majority of marked nodes in the Reactome database.

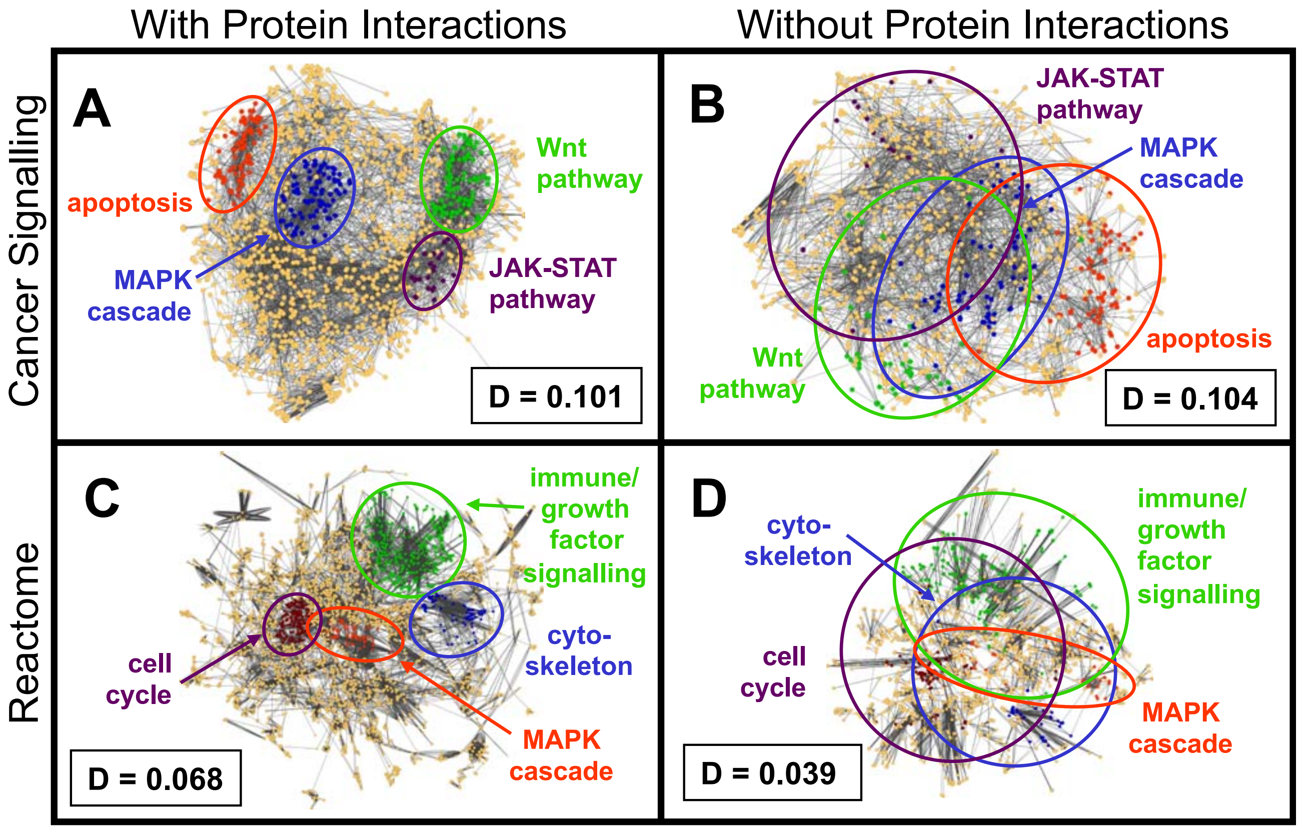

Supplementary Figure S9. Comparison of the map of human cancer signalling (A,B) and the Reactome human pathway network (C,D) EntOptLayout made with (A,C) or without the adaptor/scaffold protein interactions (B,D). Panels A and B show the map of human cancer signalling (Cui *et al.*, 2007). On Panel A all 2,403 activations, 741 inhibitions and 1,915 protein-protein interactions were visualized, while on Panel B only the activations and inhibitions were included. Panels C and D show the Reactome human pathway network (Croft *et al.*, 2011) without self-loops. On Panel C all 2681 reaction nodes (4090 edges) and 4144 protein-protein interaction nodes (13,439 edges) were visualized, while on Panel D only the reaction nodes were included. All images were visualized by the Cytoscape (Shannon *et al.*, 2003) prefuse force-directed layout option and the subsequent use of the EntOptLayout plug-in using the same settings as detailed in the legend of Fig 1. of the main text with the 'square of the adjacency matrix' option and ignoring self-loops in case of the Reactome network. “D” denotes the normalized information loss (relative entropy) of the layouts. Circled segments of the image highlight various major protein complexes (Panels A and B: blue, MAP kinase pathway, 92 nodes; green, Wnt pathway, 92 nodes; red, apoptosis, 78 nodes; purple, JAK-STAT pathway, 24 nodes. Panels C and D: blue, cytoskeleton, 104/49 nodes [all node number pairs represent panels C/D, respectively]; green, immune/growth factor signalling, 682/365 nodes; red, MAP kinase pathway, 112/63 nodes; purple, cell cycle, 249/137 nodes). Major protein complexes were identified by the consensus function of their nodes having the largest community centrality (showing the same core nodes of the respective network community as calculated by the ModuLand plug-in; Szalay-Bekő *et al.*, 2012) identifying the consensus functions of the majority of marked nodes in Uniprot (Panels A and B; The UniProt Consortium, 2017) and the Reactome database (Panels C and D).

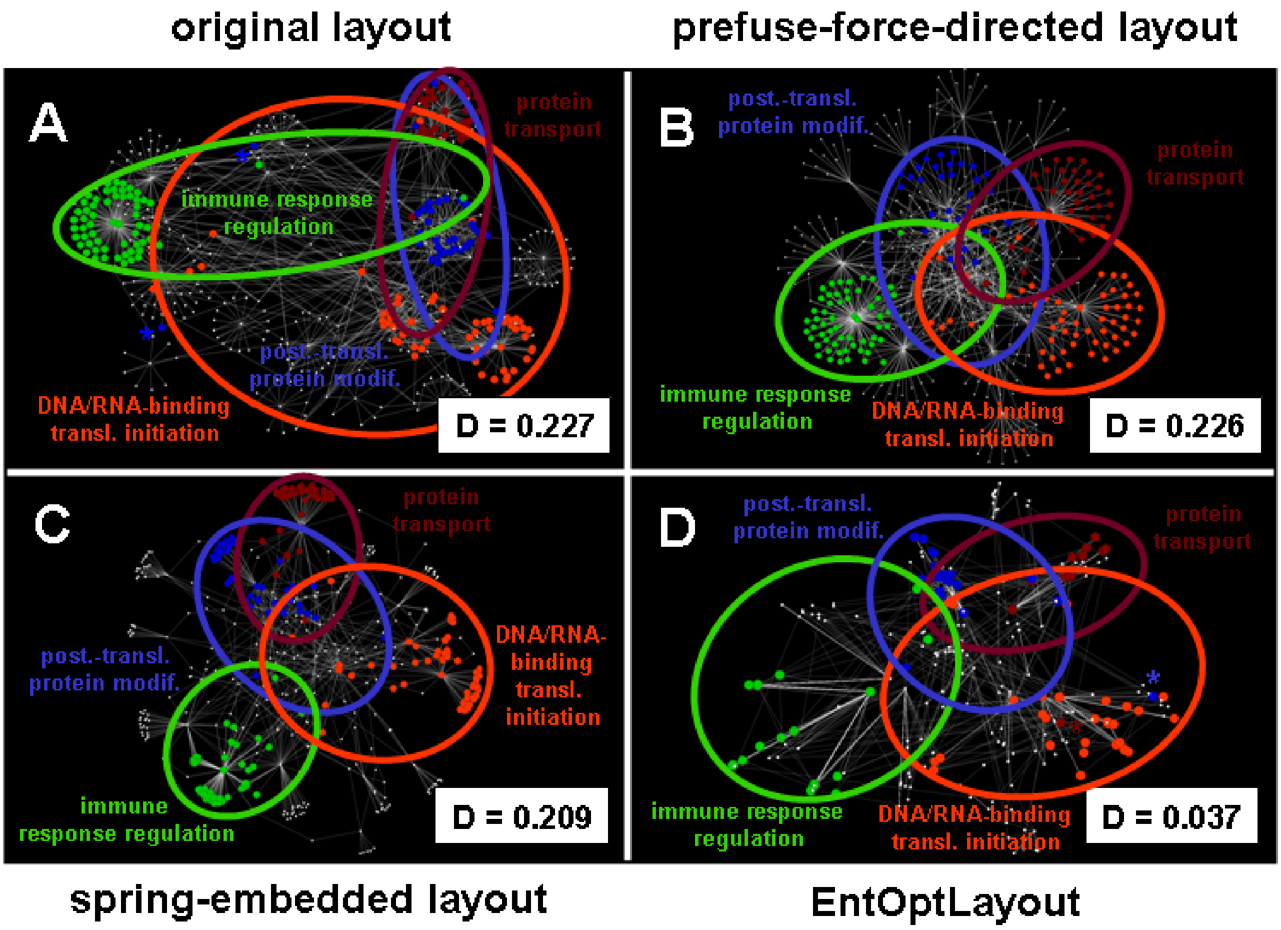

Supplementary Figure S10. Comparison of the Cytoscape welcome screen affinity purification example network original layout (A), prefuse force directed layout (B), spring-embedded layout (C) and EntOptLayout (D). Panels A through D show the Cytoscape welcome screen affinity purification example network (Morris *et al.*, 2014) visualized by the original layout (Panel A), the Cytoscape (Shannon *et al.*, 2003) prefuse force-directed layout (Panel B), spring-embedded layout (Panel C) or EntOptLayout plug-in using force-directed pre-ordering and the same settings as detailed in the legend of Fig 1. of the main text with the 'square of the adjacency matrix' option (Panel D). "D" values denote the normalized information loss (relative entropy) of the layouts (in case of the original and Cytoscape layouts node positions were imported to the EntOptLayout plug-in, and only the node probability distributions were optimized keeping the node positions intact). Circled segments of the image highlight network clusters identified by Markov Cluster Algorithm (MCL clustering; Enright *et al.* 2002; http://www.rbvi.ucsf.edu/cytoscape/clusterMaker2/#mcl). Clusters were named using a consensus function of most nodes (blue: post-translational protein modification, ubiquitination, spliceosome; purple: protein transport; green: regulation of immune response and red: DNA and RNA binding proteins, translation initiation). All the four visualizations have significant overlap of the clusters. However, the information loss is significantly smaller (4% instead of 21 to 23%) in case of the EntOptLayout than using any of the other layouts.

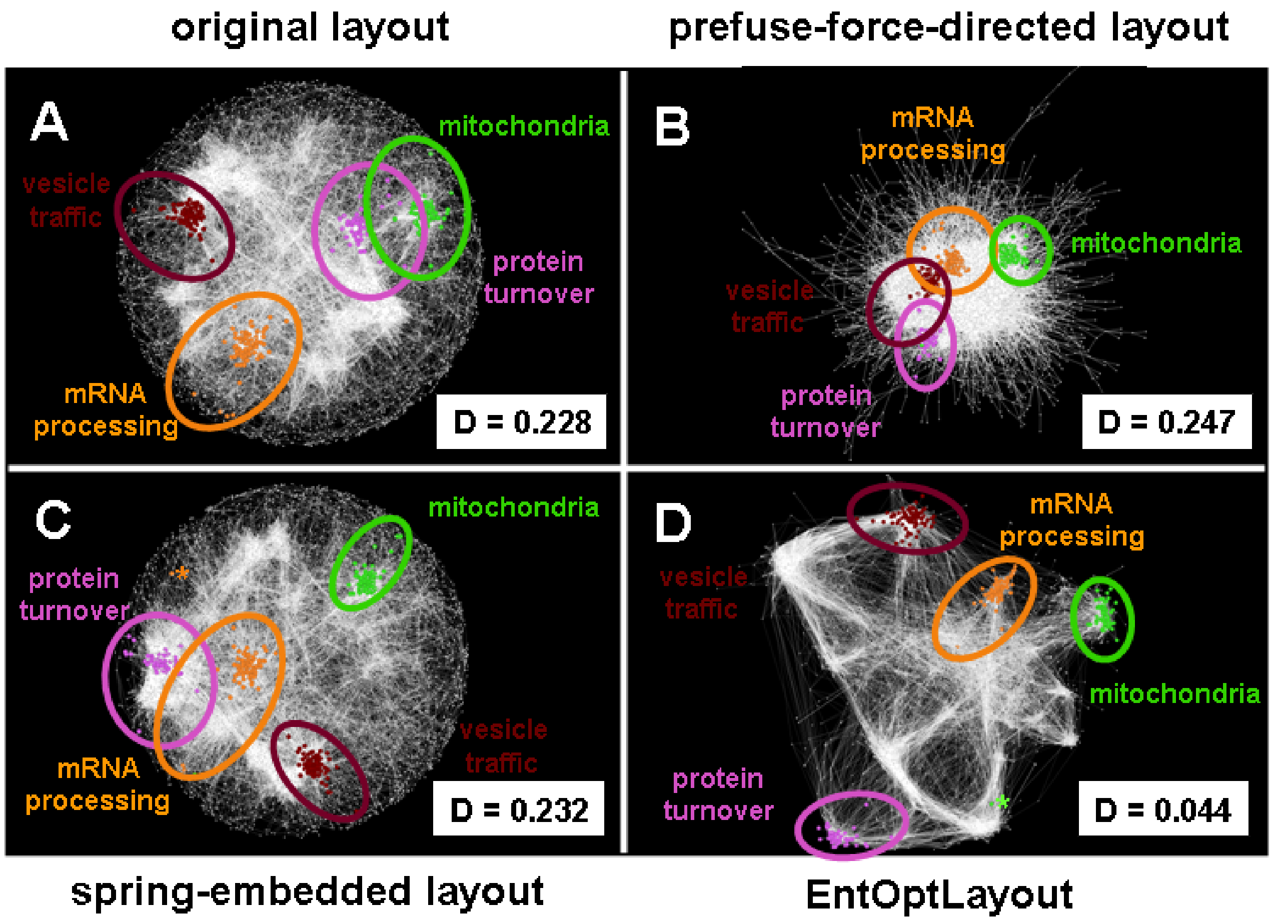

Supplementary Figure S11. Comparison of the Cytoscape welcome screen genetic interaction example network original layout (A), prefuse force directed layout (B), spring-embedded layout (C) and EntOptLayout (D). Panels A through D show the Cytoscape welcome screen genetic interaction example network (Costanzo *et al.*, 2016) visualized by the original layout (Panel A), the Cytoscape (Shannon *et al.*, 2003) prefuse force-directed layout (Panel B), spring-embedded layout (Panel C) or EntOptLayout plug-in using force-directed pre-ordering and the same settings as detailed in the legend of Fig 1. of the main text with the 'square of the adjacency matrix' option (Panel D). "D" values denote the normalized information loss (relative entropy) of the layouts (in case of the original and Cytoscape layouts node positions were imported to the EntOptLayout plug-in, and only the node probability distributions were optimized keeping the node positions intact). Circled segments of the image highlight network clusters identified by Markov Cluster Algorithm (MCL clustering; Enright *et al.* 2002, http://www.rbvi.ucsf.edu/cytoscape/clusterMaker2/#mcl). Clusters were named using a consensus function of most nodes (light-purple: protein turnover; deep-purple: vesicle traffic; green: mitochondria and orange: mRNA processing). The original layout and the two Cytoscape visualizations have overlaps of the clusters. However, with the exception of a single outlier node the four clusters are visually clearly distinct and well separated on the EntOptLayout image. In addition, the information loss is significantly smaller (4% instead of 23 to 24%) in case of the EntOptLayout than using any of the other layouts.

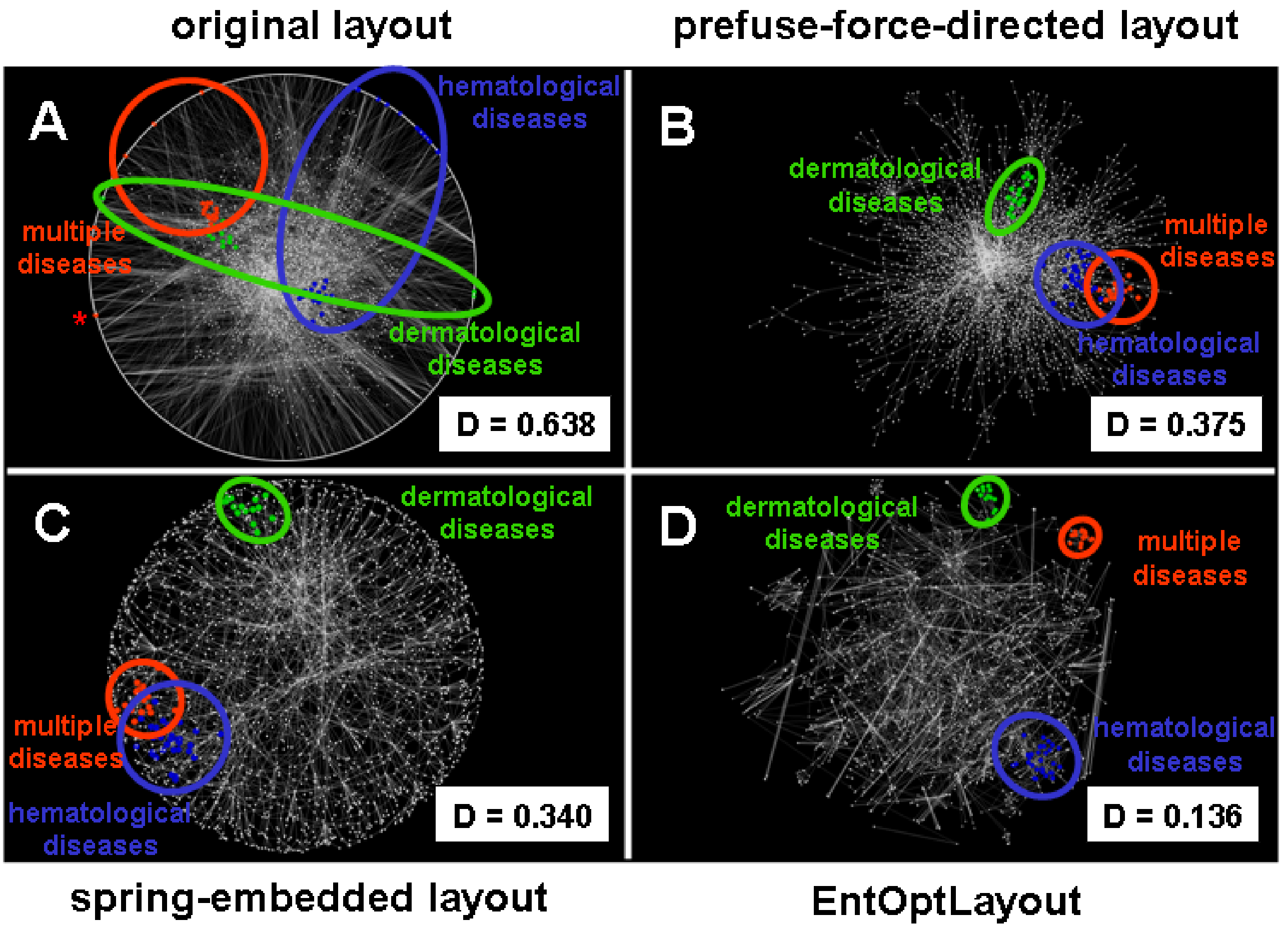

Supplementary Figure S12. Comparison of the Cytoscape welcome screen diseases example network original layout (A), prefuse force directed layout (B), spring-embedded layout (C) and EntOptLayout (D). Panels A through D show the Cytoscape welcome screen disease example network visualized by the original layout (Panel A), the Cytoscape (Shannon *et al.*, 2003) prefuse force-directed layout (Panel B), spring-embedded layout (Panel C) or EntOptLayout plug-in using force-directed pre-ordering and the same settings as detailed in the legend of Fig 1. of the main text with the 'square of the adjacency matrix' option (Panel D). "D" values denote the normalized information loss (relative entropy) of the layouts (in case of the original and Cytoscape layouts node positions were imported to the EntOptLayout plug-in, and only the node probability distributions were optimized keeping the node positions intact). Circled segments of the image highlight network clusters identified by Markov Cluster Algorithm (MCL clustering; Enright *et al.* 2002, http://www.rbvi.ucsf.edu/cytoscape/clusterMaker2/#mcl). Clusters were named using a consensus function of most nodes (blue: mainly hematological diseases; green: mainly dermatological diseases and orange: multiple diseases). The original layout and the two Cytoscape visualizations have various overlaps of the clusters. However, the three clusters are visually clearly distinct and well separated on the EntOptLayout image. In addition, the information loss is significantly smaller (14% instead of 64, 37 or 34%) in case of the EntOptLayout than using any of the other layouts.

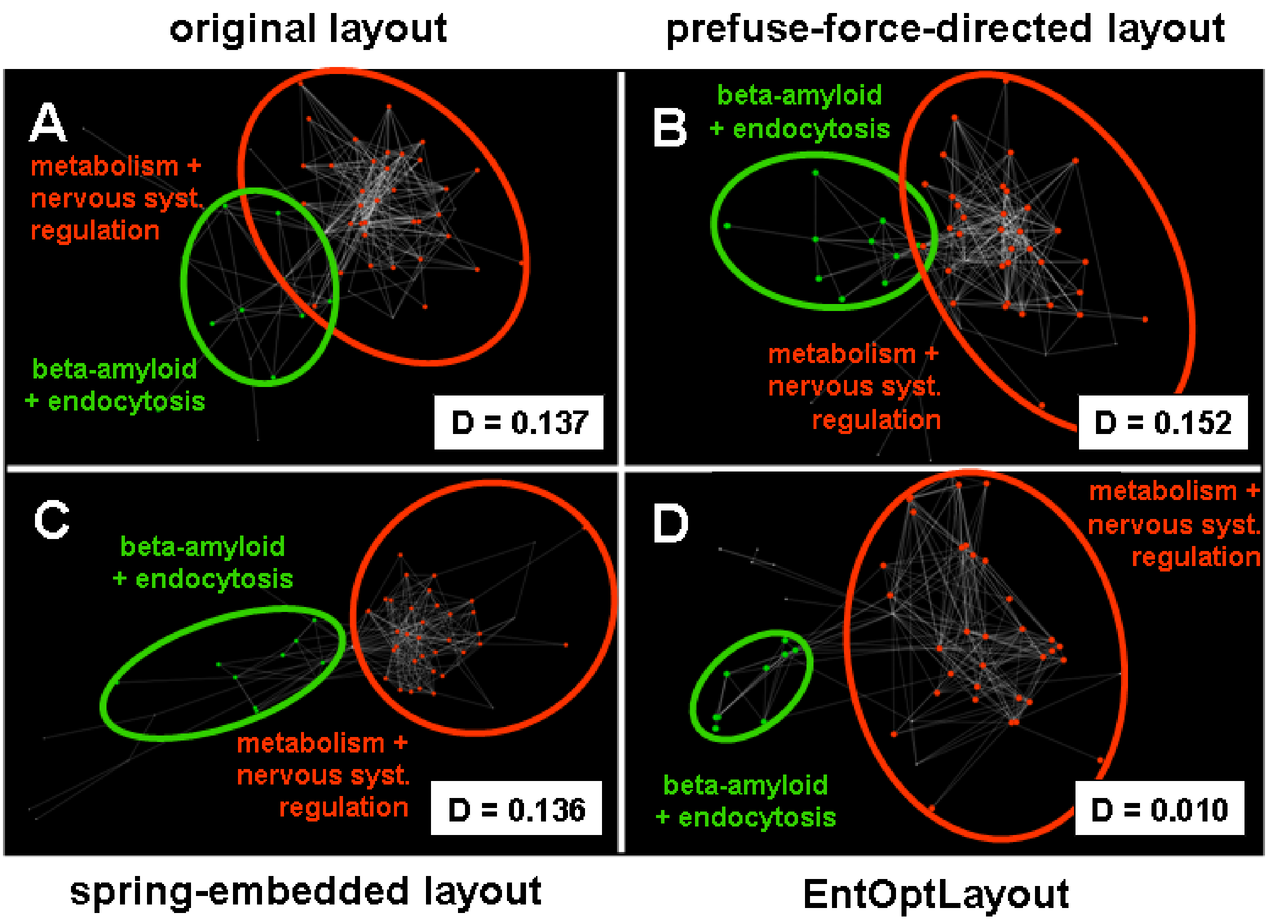

Supplementary Figure S13. Comparison of the 75 node STRING Alzheimer's disease-related protein-protein interaction network original layout (A), prefuse force directed layout (B), spring-embedded layout (C) and EntOptLayout (D). Panels A through D show an Alzheimer's disease-related protein-protein interaction network generated by the stringApp Cytoscape plug-in (Doncheva *et al.*, 2019) http://apps.cytoscape.org/apps/stringApp) downloading the top 75 disease-related nodes. The network was visualized using the original layout (Panel A), the Cytoscape (Shannon *et al.*, 2003) prefuse force-directed layout (Panel B), spring-embedded layout (Panel C) or the EntOptLayout plug-in using force-directed pre-ordering and the same settings as detailed in the legend of Fig 1. of the main text with the 'square of the adjacency matrix' option (Panel D). “D” values denote the normalized information loss (relative entropy) of the layouts (in case of the original and Cytoscape layouts node positions were imported to the EntOptLayout plug-in, and only the node probability distributions were optimized keeping the node positions intact). Circled segments of the image highlight network clusters identified by Markov Cluster Algorithm (MCL clustering; Enright *et al.* 2002, http://www.rbvi.ucsf.edu/cytoscape/clusterMaker2/#mcl). Clusters were named using a consensus function of most nodes (green: regulation of beta-amyloid formation, regulation of endocytosis and orange: regulation of metabolic process, nervous system development). The original layout and prefuse force-directed layout have an overlap of the two clusters. The two clusters do not overlap using the spring-embedded layout option. However, the two clusters are visually clearly distinct and well separated on the EntOptLayout image. In addition, the information loss is significantly smaller (1% instead of 14 to 15%) in case of the EntOptLayout than using any of the other layouts.

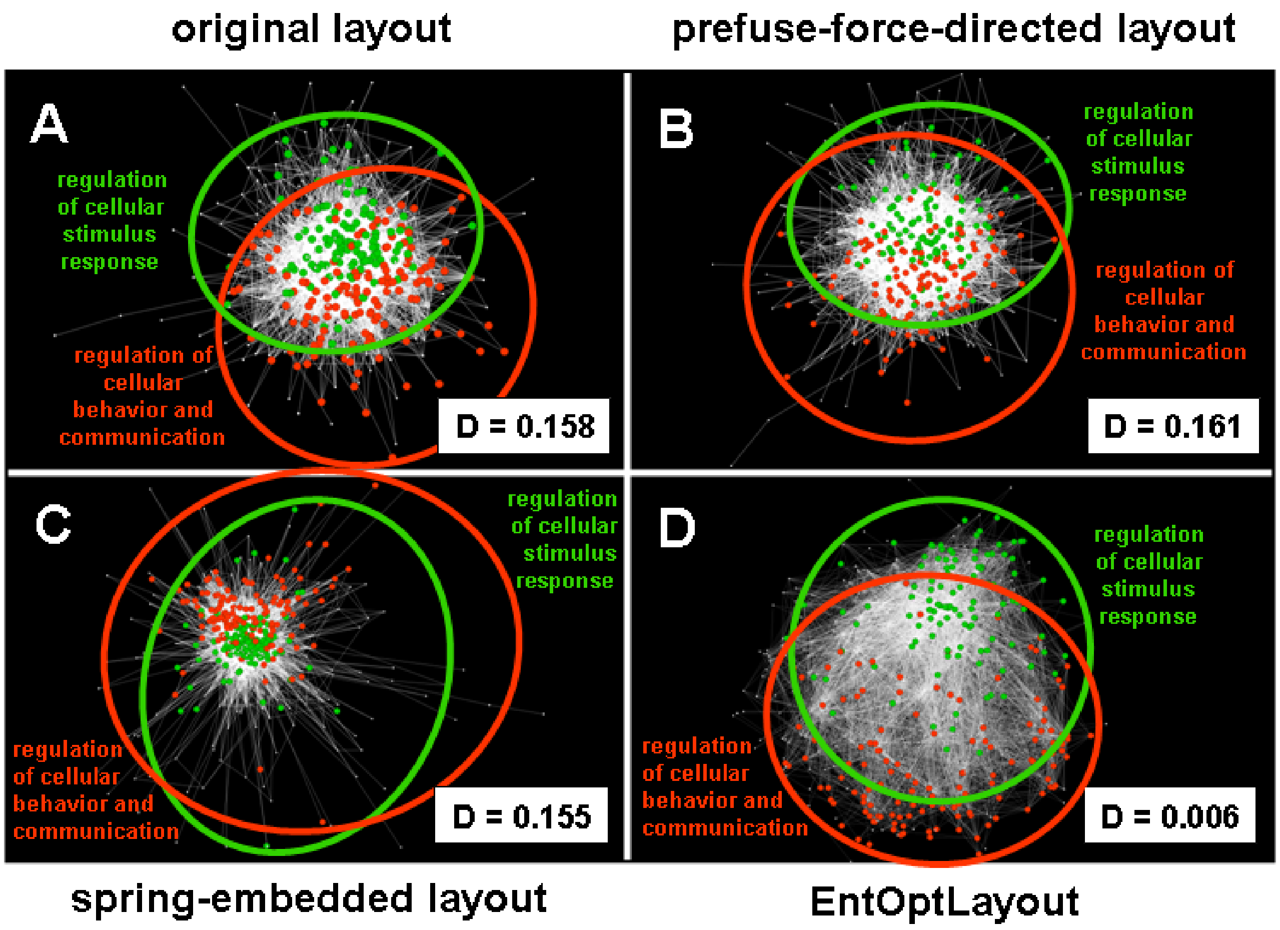

Supplementary Figure S14. Comparison of the 500 node STRING Alzheimer's disease-related protein-protein interaction network original layout (A), prefuse force directed layout (B), spring-embedded layout (C) and EntOptLayout (D). Panels A through D show an Alzheimer's disease-related protein-protein interaction network generated by the stringApp Cytoscape plug-in (Doncheva *et al.*, 2019) http://apps.cytoscape.org/apps/stringApp) downloading the top 500 disease-related nodes. The network was visualized using the original layout (Panel A), the Cytoscape (Shannon *et al.*, 2003) prefuse force-directed layout (Panel B), spring-embedded layout (Panel C) or the EntOptLayout plug-in using force-directed pre-ordering and the same settings as detailed in the legend of Fig 1. of the main text with the 'square of the adjacency matrix' option (Panel D). “D” values denote the normalized information loss (relative entropy) of the layouts (in case of the original and Cytoscape layouts node positions were imported to the EntOptLayout plug-in, and only the node probability distributions were optimized keeping the node positions intact). Circled segments of the image highlight network clusters identified by Markov Cluster Algorithm (MCL clustering; Enright *et al.* 2002, http://www.rbvi.ucsf.edu/cytoscape/clusterMaker2/#mcl). Clusters were named using a consensus function of most nodes (green: response to cellular stimulus, regulation of response to cellular stimulus and orange: regulation of cellular process, cell communication, cell-cell signalling, behaviour). All the four layouts have large overlaps of the two clusters. However, the information loss is significantly smaller (0.6% instead of 15 to 16%) in case of the EntOptLayout than using any of the other layouts.

Supplementary References

Costanzo,M. *et al.* (2016) A global genetic interaction network maps a wiring diagram of cellular function. *Science* **353**, 1381.

Croft,D., *et al*. (2011) Reactome: a database of reactions, pathways and biological processes. *Nucleic Acids Res*. **39**, D691-D697. (downloaded version v61: 17.08.02)

Cui,Q., et. al. (2007) A map of human cancer signaling. *Mol. Syst. Biol.* **3**, 152 (downloaded on 17.07.22)

Danaci,H.F. (2015) A customized force-directed layout algorithm for biological graphs. MSc thesis (http://etd.lib.metu.edu.tr/upload/12619592/index.pdf)

Doncheva,N.T., Morris,J., Gorodkin,J., Jensen.L.J. (2019) Cytoscape stringApp: Network analysis and visualization of proteomics data. *J. Proteome Res.* in press (doi: 10.1021/acs.jproteome.8b00702).

Enright.A.J.,Dongen,S.V.,Ouzounis C.A. (2002) An efficient algorithm for large-scale detection of protein families. *Nucleic Acids Res.* **30**, 1575–1584.

Hornbeck,P.V., *et al.* (2015) PhosphoSitePlus, 2014: mutations, PTMs and recalibrations. *Nucleic Acids Res*. **43**, D512-D520. (downloaded: 17.04.18)

Kovács IA, Palotai R, Szalay MS, Csermely P. (2010) Community landscapes: an integrative approach to determine overlapping network module hierarchy, identify key nodes and predict network dynamics. *PLoS One* **5**, e12528.

Kovács,I.A., *et al.* (2015) A unified data representation theory for network visualization, ordering and coarse-graining. *Sci. Rep.,* **5**, 13786.

Lancichinetti,A., Fortunato,S., Radicchi, F. (2008) Benchmark graphs for testing community detection algorithms. *Phys. Rev. E* **78**, 046110.

Mihalik,Á., Csermely,P. (2011) Heat shock partially dissociates the overlapping modules of the yeast protein-protein interaction network: a systems level model of adaptation. *PLoS Comput. Biol.* **7**, e1002187.

Morris,J.H., *et al.* (2014) Affinity purification-mass spectrometry and network analysis to understand protein-protein interactions. *Nature Protocols* **9**, 2539-2554.

Mosca,R., Céol,A., Aloy,P. (2013) Interactome3D: adding structural details to protein networks. *Nature Meth.* **10**, 47-53. (downloaded on 17.03.05)

Saad,Y. (2003) *Iterative Methods for Sparse Linear Systems*. SIAM, Philadelpha, PA, USA.

Shannon, P., *et al.* (2003) Cytoscape: a software environment for integrated models of biomolecular interaction networks. *Genome Res*. **13**, 2498-5204.

Szalay-Bekő,M., et al. (2012) ModuLand plug-in for Cytoscape: determination of hierarchical layers of overlapping network modules and community centrality. *Bioinformatics* **15**, 2202-2204.

The UniProt Consortium (2017) UniProt: the universal protein knowledgebase *Nucleic Acids Res*. **45**, D158-D169.
